# An Infrastructure-Free Indoor Localization Algorithm for Smartphones

**DOI:** 10.3390/s18103317

**Published:** 2018-10-03

**Authors:** Qu Wang, Haiyong Luo, Aidong Men, Fang Zhao, Yan Huang

**Affiliations:** 1 School of Information and Communication Engineering, Beijing University of Posts and Telecommunication, Beijing 100876, China; wangqu@ict.ac.cn (Q.W.); menad@bupt.edu.cn (A.M.); 2 Beijing Key Laboratory of Mobile Computing and Pervasive Device, Institute of Computing Technology Chinese Academy of Sciences, Beijing 100190, China; 3 School of Software Engineering, Beijing University of Posts and Telecommunication, Beijing 100876, China; zfsse@bupt.edu.cn; 4 State Key Laboratory of Advanced Optical Communication Systems and Networks, Peking University, Beijing 100871, China; huangyan0910@pku.edu.cn

**Keywords:** indoor positioning, visible light, magnetic field, fingerprints matching, smartphone

## Abstract

Accurate indoor positioning technology provides location-based service for a variety of applications. However, most existing indoor localization approaches (e.g., Wi-Fi and Bluetooth-based methods) rely heavily on positioning infrastructure, which prevents their large-scale deployment and limits the range at which they are applicable. Here, we proposed an infrastructure-free indoor positioning and tracking approach, termed LiMag, which used ubiquitous magnetic field and ambient lights (e.g., fluorescent, incandescent, and light-emitting diodes (LEDs)) without containing modulated information. We conducted an in-depth study on both the advantages and the challenges in leveraging magnetic field and ambient light intensity for indoor localization. Based on the insights from this study, we established a hybrid observation model that took full advantage of both the magnetic field and ambient light signals. To address the low discernibility of the hybrid observation model, LiMag first generated a single-step fingerprint model by vectorizing consecutive hybrid observations within each step. In order to accurately track users, a lightweight single-step tracking algorithm based on the single-step fingerprints and the particle filter framework was designed. LiMag leveraged the walking information of users and several single-step fingerprints to generate long trajectory fingerprints that exhibited much higher location differentiation ability than the single-step fingerprint. To accelerate particle convergence and eliminate the accumulative error of single-step tracking algorithm, a long trajectory calibration scheme based on long trajectory fingerprints was also introduced. An undirected weighted graph model was constructed to decrease the computational overhead resulting from this long trajectory matching. In addition to typical indoor scenarios including offices, shopping malls and parking lots, we also conducted experiments in more challenging scenarios, including large open-plan areas as well as environments characterized by strong sunlight. Our proposed algorithm achieved a 75th percentile localization accuracy of 1.8 m and 2.2 m, respectively, in the office and shopping mall tested. In conclusion, our LiMag algorithm provided location-based service of infrastructure-free with significantly improved localization accuracy and coverage, as well as satisfactory robustness inside complex indoor environments.

## 1. Introduction

Accurate and pervasive indoor positioning significantly facilitates of our daily life tasks [1]. For example, localization and navigation services in shopping malls, office buildings or parking garages; security domain applications for anti-terrorism action, emergency rescue and exploration missions; consumer analytics through aggregated foot-traffic patterns and dwell time; product recommendation and coupon delivery in retail stores. A recent market report predicted that the global indoor location market size is expected to grow from USD 7.11 billion in 2017 to USD 40.99 billion by 2022, at a compound annual growth rate (CAGR) of 42.0% during the forecast period [2].

In order to meet this explosive demand, various indoor positioning approaches have recently been developed, including Wi-Fi [3,4], UWB [5], BLE [6] and visual methods [7]. However, these methods are costly and ineffective when the radio signal is weak or not available in some scenarios, such as underground parking lots. Moreover, positioning technology based on radio frequency (RF) is prohibited in electromagnetically sensitive environments such as hospitals, airplanes or mines, due to safety concerns [8].

Various lighting infrastructures have been widely installed in indoor environments to provide ubiquitous illumination services. Visible light is susceptible to the propagation environment features, such as shadowing, scattering, and reflection from different surfaces, which cause obvious changes in light intensity at different locations. The propagation of light is however not affected by electromagnetic interference. In addition, the received power of optical signals is more stable than the received power of radio signals [9]. Thus, using visible light instead of radio allows for higher indoor positioning accuracy. Moreover, visible light is used for illumination while allowing for indoor positioning. Therefore, visible light positioning (VLP) is becoming a promising positioning technology [10,11,12,13].

Most VLP studies were based on the propagation model of light [8,12,14] and imaging geometry relations [11] using modulated smart LEDs as beacons. However, high precision VLP schemes often require well-shaped LEDs [8,12,14,15] (for propagation modeling) or ultra-dense deployment [12,16] (multiple LEDs within the camera field of view), which significantly limit their application. Moreover, most existing VLP systems leverage specialized “smart beaconing LEDs” (working at specific flicker frequency by customized control circuits) as localization beacon for target estimation, are incompatible with the current lighting infrastructures. Unfortunately, fluorescent lights (FLs) currently occupy 85% of the commercial buildings [17]. In comparison, essential LED lights account for only 12%, with a predicted market domination in approx. 10 to 15 years [17], not to mention the scarcity of smart beaconing LEDs. Few studies based on existing illumination infrastructures and commercial smartphones achieve high accuracy in specific scenarios.

Jimenez et al. [18] presented a light-matching-based indoor localization method using the position, orientation and shape information of lamps, and modeled illumination intensity using an inverse-square law to estimate user positions. Their system relies on the asymmetries/irregularities of luminary placement to distinguish different luminaries, which results in feeble anti-interference ability. Furthermore, the localization performance of light-matching was only evaluated via simulation, and its performance in real-world environments remained unknown. Zhang et al. introduced LiTell [19], which utilizes unmodified fluorescent lights (FLs) and a commercial smartphone for indoor positioning. LiTell only works for FLs, and is unsuitable for LEDs and incandescent lamps. More importantly, the cameras of commercial smartphones are characterized by a low dynamic range, which limits the working range of LiTell to around 2.5 m, as a result of which many blind areas might be generated. The mechanism of multi-light matching results in high processing latency (>2 s) and high power consumption (>2.7 watts). The most problematic feature of LiTell is its use of the back-facing camera. Together, these factors severely limit its application in a real-world environment. Zhu et al. designed a different algorithm called iLAMP [20], which uses the conventional LEDs and fluorescent lamps found inside today’s buildings for VLP by extracting the spatial radiance pattern (SRP) of lamps. However, frequent SRP extraction inevitably results in high power consumption. While power consumption is significantly lower for iLAMP (0.93 watts) compared to LiTell (2.7 watts), it still remains too high to be suitable for smartphones. Zhao et al. proposed NaviLight [21], which uses visible light fingerprints and KNN to achieve position estimation using existing lighting infrastructure without containing modulated information. NaviLight achieves sub-meter localization accuracy, under the premise of keeping smartphone attitude constant. However, it is challenging to maintain smartphone attitude constant and heading consistent with walking direction. Moreover, NaviLight fails to perform in an environment characterized by strong sunlight, such as a building with lots of windows or skylights, because sunlight severely affects fingerprints based on visible light. The most significant drawback of existing VLP systems is the fact that they are vulnerable to sunlight noise, as the luminance of a lamp is hundreds of times lower than that of sunlight [22].

The Earth’s magnetic field [23] is omnipresent and susceptible to be affected by many factors, such as steel-reinforced concrete, metallic door frames, pillars, furniture, electronic equipment, appliances. These factors generate stable indoor magnetic anomalies that differ across locations. The magnetic field has the potential to localize and track users because of its location-related, infrastructure-free, and energy efficient features [24]. The Earth’s magnetic field has been considered for indoor positioning in many studies [1,24,25,26,27]. However, several excellent reviews have commented that the magnitude of magnetism is ambiguous in large indoor spaces or non-steel structure buildings [28,29,30,31].

Indoor positioning technologies based on the magnetic field have attracted considerable interest because magnetic sensors have become an essential sensor in most mobile devices. SemanticSLAM [32] extracts indoor magnetic field anomalies using an unsupervised-learning approach to identify locations. Another study designed a system that uses ambient magnetic field anomalies to build magnetic maps for indoor localization in [33]. To estimate target position, MaLoc [25] fuses the inertial sensor data and step counting, heading change and hybrid magnetic measurement between two contiguous steps within a novel reliability-augmented particle filter framework. LocateMe [34] leverages sequences of magnetic readings to estimate a target’s position using smartphones. However, it only achieves a room level localization accuracy. In addition, it is restricted in one-dimensional environments, such as a corridor. To estimate the target’s positions, GROPING [35] leverages crowdsourcing-based fingerprints map and the DTW algorithm based on a revised Monte Carlo model using the magnetic signal. Luo et al. [36] also proposed an algorithm for the automatic construction of an indoor floor plan, together with a magnetic fingerprints map of unmapped buildings using crowdsourced smartphone data. Fusion of magnetic field and other location sources is a widely considered in indoor positioning. Magicol [1] integrates magnetic field and Wi-Fi data to deal with the low discernibility of the magnetic field, which is designed with a particle filter-based inertial measurement unit (IMU) engine for localization and tracking. VMag [37] utilizes a neural-network-based method to extract deep visual features of visual image and designs a context-aware particle filtering framework to fuse the magnetic field and deep visual features. These magnetic positioning methods have already achieved a high positioning accuracy, but it only applies to the areas with route constraints, e.g., long and narrow corridors.

**Figure 1:**
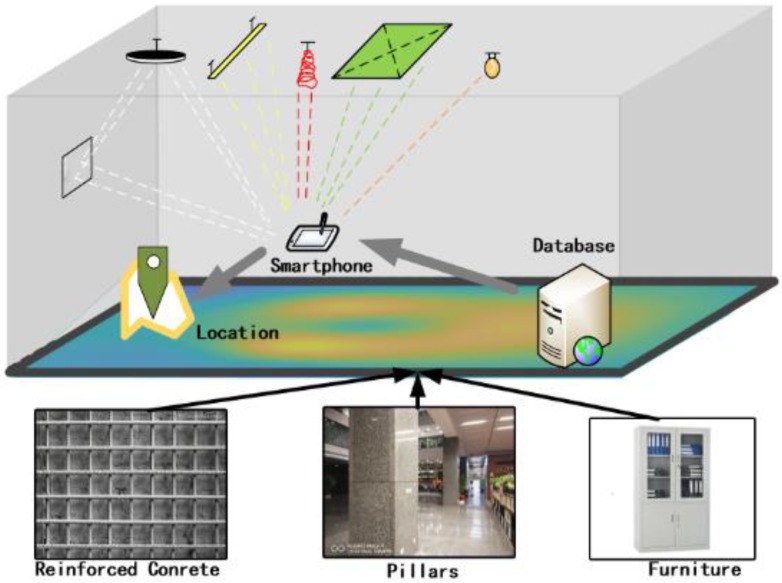
The working scenario of LiMag.

As shown in Figure 1, we designed and evaluated LiMag—a specialized infrastructure-free indoor positioning algorithm based on a smartphone using ambient ***Li***ght sources (e.g., fluorescent, incandescent, and LEDs) and ***Mag***netic field data with a trajectory matching approach. LiMag extracts the hybrid features of ambient light and magnetic field signals to establish a hybrid observation model, then several hybrid observations within one step or multi-steps constitute a hybrid fingerprints model (HFM) as a location-specific signature. The HFM not only alleviates sunlight interference but also enhances the discernibility of the open-plan area. LiMag performs a single-step tracking algorithm based on particle filter framework and long trajectory calibration scheme using HFM and an undirected weighted graph model (UWGM) [38] to provide an energy efficient location-service.

To accomplish an infrastructure-free and accurate localization for complex indoor environments, we must address the following challenges: (1) It is difficult to distinguish different locations using only light or magnetic signals as we may find many locations with same magnetic field magnitude in the rather short trace. Mobile objects also have a considerable impact on the propagation of light. Therefore, a single observed value at a fixed position is insufficient to serve as a reliable and unique location signature. (2) The indoor environment is complex and various. Individual buildings may install sidewall windows or skylights through which sunlight will severely disturb the light sensor readings. Magnetic field signals fluctuate with feebleness in large indoor open-plan areas or non-steel structure building. (3) It remains challenging to quickly match fingerprints collected online with those pre-collected offline.

To address these challenges: (1) We collected the mixed signal of ambient light and magnetic, and leveraged user motion to vectorize several hybrid observations to form a higher discernibility signature, including single-step trajectory and long trajectory fingerprints. (2) We extracted the hybrid features of magnetic field signals and ambient light to establish a hybrid observation model that utilizes the complementary nature of the magnetic field and light intensity signals. (3) We added directional information to single-step fingerprints and trajectory fingerprint to reduce the matching complexity and improve matching accuracy. We designed LiMag to collect hybrid fingerprints and matches itself with the corresponding directed fingerprints in the pre-established hybrid map, a location database built in offline stage with mappings between light intensity and magnetic values and their locations, to estimate the user’s position, leveraging a subsequent dynamic time warping (DTW) algorithm [39] and UWGM. The key contributions of our study are as follows:

- We performed an in-depth study of both the advantageous properties and the challenges in leveraging the magnetic field and ambient light intensity for indoor localization. Based on these studies, we extracted the hybrid features of indoor ambient light and magnetic field signals within one or several steps to construct a hybrid fingerprint model as a location-specific signature.
- We designed a long trajectory calibration scheme based on an undirected weighted graph model. The undirected weighted graph model was constructed to reduce the computational overhead resulting from long trajectory matching (LTM. A result validation of trajectory matching was also designed to filter the DTW output and identify which LTM result is accurate. Lastly, the heading and step length were calibrated by the matched trajectory.
- We proposed a single-step tracking algorithm based on the hybrid fingerprints model and the particle filter framework. The weight of particle filters was updated by the hybrid fingerprints model that not only alleviates sunlight interference but also enhances the location differentiation ability in large open-plan areas. The cumulative error of the particle filters was calibrated by the result of the long trajectory calibration scheme.
- We implemented LiMag entirely on the Android platform and conducted extensive experiments in multiple scenarios, including a large open-plan area as well as an environment characterized by strong sunlight, and the results showed that LiMag achieved an accuracy of 1.8∼3.5 m in typical indoor scenarios.

This paper is organized as follows: in Section 2, we review the background and our empirical studies of ambient light intensity and magnetic field. In Section 3, we detail the proposed the infrastructure-free hybrid indoor localization algorithm based on a smartphone. In Section 4, we evaluate the proposed scheme, and in Section 5, we provide conclusions that summarize the importance of our work for specialized infrastructure-free hybrid indoor localization.

## 2. Insights on Visible Light and Magnetic Fields

In this section, we conducted extensive experiments in a real environment to investigate whether the hybrid signal of visible light and magnetic field is stable and discriminative enough to act as a suitable location signature.

### 2.1. Favorable Properties and Challenges of Visible Light

#### 2.1.1. Favorable Properties

**Figure 2:**
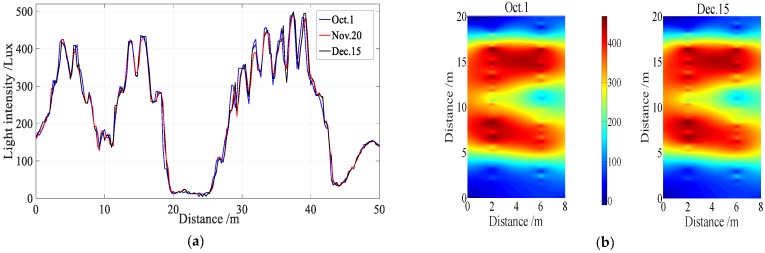
The temporal stability of light intensity. (**a**) Light intensity readings captured at different dates along the same path; (**b**) Light field map captured by smartphone over two months.

To verify the temporal stability of light signals, we walked along the same path in an office building at different times and on different days that were three months apart to collect light signals using a commercial smartphone (Huawei Mate 9). As shown in Figure 2a, the variation trends of three curves were nearly equal. We also generated a light intensity map using a smartphone in a 20 m × 8 m indoor area over two months. As shown in Figure 2b, the light signals maps of different months were similar, although the light intensity differed across locations. These experiments indicate that light intensity at the same place is stable over time under the condition that the lamp layout and architecture topological structure remains unchanged.

**Figure 3:**
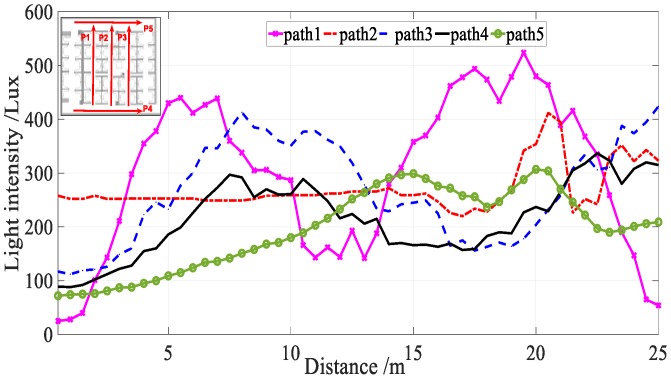
The location differentiation ability of light signals.

In addition to temporal stability, visible light has stronger location differentiation ability at different locations due to the following factors: first, lamps are often (possibly) covered with lampshades which are made from different materials and irregular decorative panels, as well as inevitable manufacturing variations. Second, reflection, refraction, scattering, and diffraction are present in the process of light propagation, and different surfaces have different light reflection factors. Therefore, the light intensity produced even by a single source is a non-uniform distribution. Moreover, multiple types of lamps may exist simultaneously, thus leading to an even more complicated light intensity distribution. As shown in Figure 3, the light intensity sequences on different paths showed evident differences.

#### 2.1.2. Challenges

**Figure 4:**
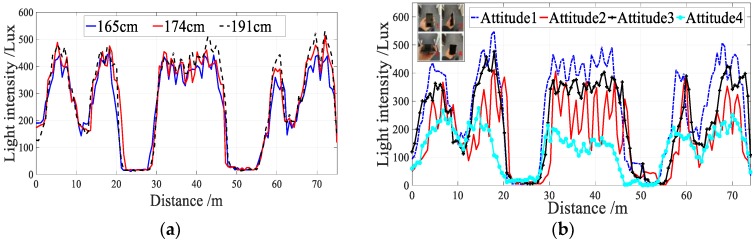
User Diversity. (**a**) Different heights; (**b**) Different attitudes.

The propagation of light is assumed to follow a Lambertian radiation pattern [40]. Therefore, the received light intensity is not only a function of the distance between receiver and beacons, but also depends on the irradiation angle and incidence angle. Figure 4a shows the sensor readings that were precisely collected by three individuals of different height (165, 174, 191 cm) along the same pa. In addition, we also collected sensor readings in different attitudes precisely along the same path using the same device and same people (see Figure 4b). From Figure 4a,b, we see that height has little effect on light intensity, but the attitude has a considerable impact.

**Figure 5:**
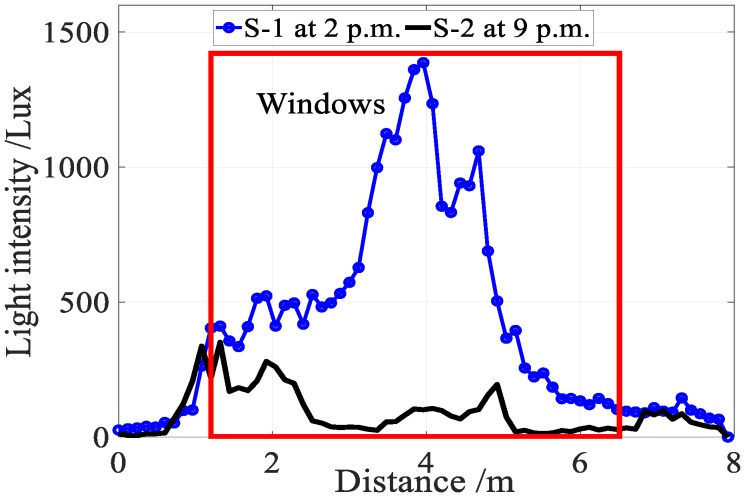
Sunlight disturbs light sensor readings obviously.

In addition to user diversity, sunlight severely disturbs the light sensor readings. As shown in Figure 5, the intensity of sunlight is several times than that of the indoor lamps, thus resulting in that the light sensor readings are unable to reflect the property of indoor lamps in the area near the windows or skylights. Damage or turned off individual lamps as well as lamp layout changes also cause a significant signal fluctuation, leading to a large localization error.

### 2.2. Favorable Properties and Challenges of Magnetic Field

#### 2.2.1. Favorable Properties

Indoor magnetic fields exhibit certain anomalies due to the disturbances caused by building construction materials and electrical appliances [1]. As long as the internal layout remains unchanged, the magnetic anomalies are stable over time [1,26,30,34,41]. Therefore, the magnetic anomalies have the potential for accurate and pervasive indoor positioning without depending on any infrastructure [26,42].

#### 2.2.2. Challenges

**Figure 6:**
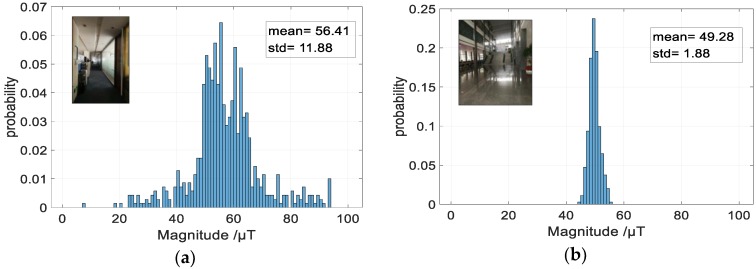
Static magnetometer measurement distribution of different scenarios by the same smartphone. (**a**) corridor; (**b**) large open-plan area.

The most problematic feature of the magnetic field is its low discernibility [1,26,34]. Construction materials and electrical appliances have little influence on the magnetic field values even at a distance of a few meters [1]. The discernibility of the magnetic field is limited, commonly within a few tens of μT. To demonstrate the discernibility of the magnetic field, we measured the magnitude of the magnetic field at 800 different locations in a corridor, and generated a histogram. We conducted the same experiments in a large open-plan area (600 $m^{2}$). The distances from the observation point to the interference sources in the corridor is smaller than that in open-area. As shown in Figure 6, the deviation of the magnetic field observations in a large open-plan area is less than that in the corridor. In other words, the discernibility of the magnetic field in the large open-plan area is less than that in the corridor. Studies [28,29,30] also demonstrates this conclusion.

A magnetometer outputs three-axis vectors (*m_x_*, *m_y_* and *m_z_*), it is natural to think of using the three-dimensional values to improve the discernibility of the magnetic signal. However, in practice, it is hard to use all of the three-axis magnetic vectors because the carrier frame of the magnetometer may change frequently and is very difficult to align with the global frame and the carrier frame [42]. To eliminate the influence of three-axis magnetic signal fluctuation with the smartphone attitude, reference [27] estimated smartphone orientation and transformed the attitude-specific three-axis raw magnetic observation of the carrier frame (b frame, i.e., smartphone) to a uniform three-axis magnetic fingerprint in the navigation frame. However, such a transformation is error-prone and inaccurate because orientation estimation usually contains errors. It is challenging to use only the magnetic field for indoor localization in large open-plan areas or non-steel structure buildings.

### 2.3. Fusion of Light Intensity and Magnetic Field

Visible light has stronger location differentiation ability at different locations, but light intensities are susceptible to environmental changes, while the magnetic field has weaker location differentiation ability in a large open-plan area and is robust against environmental changes. The complementary nature of light intensity and magnetic field signals is the fundamental reason that we combine light and magnetic signals to improve the uniqueness and robustness of location signatures.

To verify the location differentiation ability of the light intensity signal, magnetic field signal and the hybrid signal of the magnetic field and light intensity, we utilized a confusion matrix M to measure the location differentiation ability of N different locations, as shown in Equations (1) and (2):

(1) $$M=\begin{array}{c} \begin{array}{l} L_{1} \end{array}\\ \vdots \\ L_{i}\\ \begin{array}{l} \vdots \\ L_{n} \end{array} \end{array}\begin{array}{c} \begin{array}{cccc} L_{1} & \cdots & L_{i} & \begin{array}{cc} \cdots & L_{n} \end{array} \end{array}\\ \left[\begin{array}{c} \begin{array}{cccc} d_{1,1} & \cdots & d_{1,j} & \begin{array}{cc} \cdots & d_{1,n} \end{array}\\ \vdots & \ddots & \vdots & \begin{array}{cc} ⋰ & \vdots \end{array}\\ d_{i,1} & \cdots & d_{i,j} & \begin{array}{cc} \cdots & d_{i,n} \end{array}\\ \vdots & ⋰ & \vdots & \begin{array}{cc} \ddots & \vdots \end{array} \end{array}\\ \begin{array}{cccc} d_{n,1} & \cdots & d_{n,j} & \begin{array}{cc} \cdots & d_{n,n} \end{array} \end{array} \end{array}\right] \end{array}$$

(2) $$d_{i,j}={\left\|S_{i}-S_{j}\right\|}_{2},0\leq d_{i,j}\leq 1$$

where *L_i_* denotes the position of the obtained visible light and magnetic signals. The *i^th^* row and *j^th^* column of the matrix *M* represents the normalized Euclidean distance *d_i,j_* between the two signal vectors of locations *i* and *j*. *S* represents the light intensity signal, the magnetic field signal, or the hybrid signal of light intensity and magnetic field, respectively.

**Figure 7:**
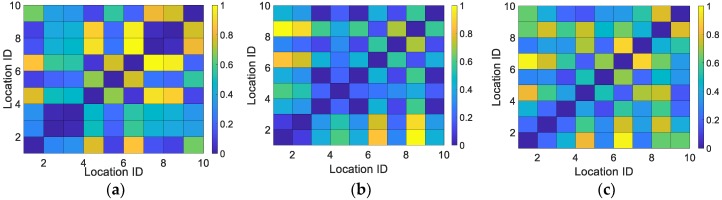
Confusion matrixes of normalized Euclidean distance. (**a**) light signal; (**b**) magnetic signal; (**c**) light and magnetic signal.

We collected the light intensity and magnetic field signal at 10 positions apart 1 m from each other in a large open-plan area, generated normalized confusion matrixes (see Figure 7). Figure 7 indicates that the location differentiation ability of the hybrid signal is better than that of the magnetic field or light intensity only.

## 3. Positioning Model

### 3.1. System Architecture

**Figure 8:**
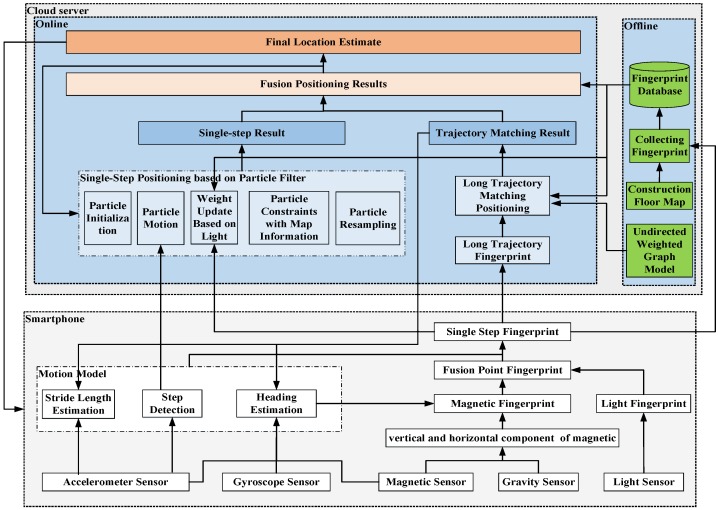
The architecture of LiMag.

As shown in Figure 8, LiMag employs the existing lighting infrastructures and magnetic field, cloud server and smartphone with light sensor, magnetometer, gyroscope and accelerometer for target localization. To reduce the energy- and resource-consumption of the smartphone, a single-step tracking algorithm based on particle filter and long trajectory calibration scheme is deployed on a cloud server. The single-step tracking algorithm based on particle filter framework continuously tracks a user through a pedestrian motion model and the HFM. The pedestrian motion model detects steps, as well as estimates the stride length and walking direction based on the observations from inertial sensor embedded in smartphone, and drives particle state update. The long trajectory calibration scheme based on UWGM provides opportunistic positioning result with high accuracy. The opportunistic positioning result is used to accelerate the particle convergence and calibrate the results of the single-step tracking algorithm.

### 3.2. Pedestrian Motion Model

#### 3.2.1. Precise Pedestrian Heading Estimation Based on Historical Information

Precise heading estimation is significant for particle motion and LTM, however, it is challenging to precisely estimate walking directions by smartphone-embedded IMU sensors (gyroscope and accelerometer) and magnetometer, due to the complicated walking patterns and indoor electromagnetism interference [43]. An improved heading estimation algorithm proposed by Wonho Kang is applied here [44]. The fused heading is estimated as follows:

(3) $$\{\begin{array}{ll} \theta_{k}=\alpha_{1}\theta_{k-1}+\beta_{1}\theta_{m,k}+\gamma_{1}\theta_{g,k}, & \theta_{\Delta ,c}\leq \theta_{\tau ,c},\theta_{\Delta ,m}\leq \theta_{\tau ,m}\\ \theta_{k}=\alpha_{2}\theta_{k-1}+\beta_{2}\theta_{m,k}+\gamma_{2}\theta_{g,k}, & \theta_{\Delta ,c}\leq \theta_{\tau ,c},\theta_{\Delta ,m}>\theta_{\tau ,m}\\ \theta_{k}=\alpha_{3}\theta_{k-1}+\beta_{3}\theta_{m,k}+\gamma_{3}\theta_{g,k}, & \theta_{\Delta ,c}>\theta_{\tau ,c},\theta_{\Delta ,m}\leq \theta_{\tau ,m},\theta_{\Delta ,g}<\theta_{\tau ,g}\\ \theta_{k}=\alpha_{4}\theta_{k-1}+\beta_{4}\theta_{m,k}+\gamma_{4}\theta_{g,k}, & \theta_{\Delta ,c}>\theta_{\tau ,c},\theta_{\Delta ,m}\leq \theta_{\tau ,m},\theta_{\Delta ,g}\geq \theta_{\tau ,g}\\ \theta_{k}=\alpha_{5}\theta_{k-1}+\beta_{5}\theta_{m,k}+\gamma_{5}\theta_{g,k}, & \theta_{\Delta ,c}>\theta_{\tau ,c},\theta_{\Delta ,m}>\theta_{\tau ,m},\theta_{\Delta ,g}<\theta_{\tau ,g}\\ \theta_{k}=\theta_{g,k}, & \theta_{\Delta ,c}>\theta_{\tau ,c},\theta_{\Delta ,m}>\theta_{\tau ,m},\theta_{\Delta ,g}\geq \theta_{\tau ,g} \end{array}$$

where *θ_k_* and *θ_k_*_−1_ represent the direction of current step and last step, respectively. *θ_m,k_* and *θ_g,k_* represent the direction of magnetic and gyroscope, respectively. *θ*_Δ,*c*_ represents the difference between *θ_m,k_* and *θ_g,k_*. *θ*_Δ,*m*_ represents the difference between *θ_m,k_* and *θ_m,k_*_−1_. *θ*_Δ,*g*_ represents the difference between *θ_g,k_* and *θ_g,k_*_−1_. *α_i_*, *β_I_* and *γ_i_* represent the weights of *θ_k_*_−1_, *θ_m,k_* and *θ_g,k_*. *θ_τ,c_*, *θ_τ,m_* and *θ_τ,g_* represent threshold parameters.

#### 3.2.2. Step Counting and Length Estimation

**Figure 9:**
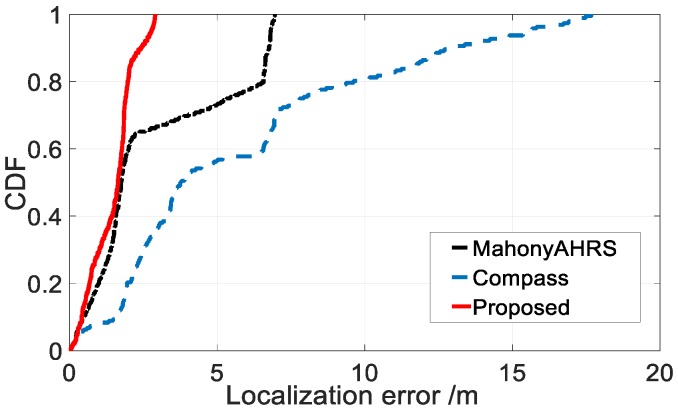
Localization accuracy comparison of PDR using different heading estimation algorithms.

To improve the robustness of the step counting, we utilized the step counting algorithm proposed by Kourosh [45], which solves the overcounting problem caused by false walking (e.g., when users use their phones for playing games in a still state). To provide more robust step length estimation, we employed a probabilistic context-aware (stationary, walking, walking sideways, climbing and descending stairs, and running) step length estimation algorithm proposed by Martinelli [46]. The performance of a PDR algorithm using the proposed heading estimation method was evaluated as Figure 9 shows.

We walked for 192 steps (including three turns), which corresponds to about 120 m in length. For each step, we calculated the Euclidean distance error between the estimated position and the real position using different heading estimation algorithms (compass, mahonyAHRS and our proposed method). Compared with the other two heading estimation methods, using our proposed heading estimation algorithm achieved higher positioning accuracy.

### 3.3 Hybrid Fingerprints Model

When the vertical direction of smartphone was reliably estimated by the 3-axis gravity sensor built in smartphone, we extracted the vertical component $m_{v}$ and horizontal component $m_{h}$ of the magnetic field vector m [41]. Then, we constructed a three-dimensional magnetic observation (vertical component $m_{v}$, horizontal component $m_{h}$, and instantaneous direction θ). We established a hybrid observation model by combining the three-dimensional magnetic observation and light intensity information. The single hybrid observation is susceptible to sensor noise, thus resulting in an inaccurate localization result. To reduce the occasional fluctuation caused by sensor noise, we leveraged the walking information to vectorize several hybrid observations into hybrid fingerprints of high dimensional. Here, we constructed two types of hybrid fingerprints: single-step fingerprints and long trajectory fingerprints, which were used for user tracking and calibration, respectively.

#### 3.3.1 Single-Step Fingerprints

Increasing the spatial coverage of observations is an efficient way to improve the discernibility of signals. Therefore, we define the vector of multiple hybrid observations between within consecutive steps as single-step fingerprints. Magnetic field and light intensity are collected between two consecutive steps, and automatically associates with the position of each step. Therefore, the single-step hybrid fingerprint s is described in Equation (4).

(4) $$s_{i}=\{ID_{i},(x_{i},y_{i}),\theta_{i},\left[\begin{array}{c} \begin{array}{cccc} m_{v1} & \cdots & m_{vk} & \begin{array}{cc} \cdots & m_{vn} \end{array} \end{array}\\ \begin{array}{cccc} m_{h1} & \cdots & m_{hk} & \begin{array}{cc} \cdots & m_{hn} \end{array} \end{array}\\ \begin{array}{cccc} \theta_{1} & \cdots & \theta_{k} & \begin{array}{cc} \cdots & \theta_{n} \end{array} \end{array}\\ \begin{array}{cccc} l_{1} & \cdots & l_{k} & \begin{array}{cc} \cdots & l_{n} \end{array} \end{array} \end{array}\right]\}$$

where $\left[m_{v1},\cdots ,m_{vk},\cdots ,m_{vn}\right]$, $\left[m_{h1},\cdots ,m_{hk},\cdots ,m_{hn}\right]$, $\left[\theta_{1},\cdots ,\theta_{k},\cdots ,\theta_{n}\right]$ and $\left[l_{1},\cdots ,l_{k},\cdots ,l_{n}\right]$ represent the vertical and horizontal component of the magnetic field, instantaneous direction and light intensity observation sequence between two consecutive steps, respectively. $ID_{i}$ represents a global step identifier. $\left(x_{i},y_{i}\right)$ is the central position of one step. $\theta_{i}$ is the step direction. To eliminate the occasional fluctuation caused by user swaying sideways and other noises, we regard the average value of instantaneous direction observation sequence between two consecutive steps as the step direction.

#### 3.3.2 Long Trajectory Fingerprints

We define the path between two turns as an atomic path. To further enhance the discernibility of hybrid signals, we combine all single-step fingerprints in atomic path into long trajectory fingerprints. Considering that pedestrians tend to walk in a straight line, we obtain a more precise trajectory direction by calculating the direction average of all single-step fingerprints in same atomic path.

Therefore, the long trajectory fingerprint t is described as Equation (5).

(5) $$t_{j}=\left\{TID_{j},T\theta_{j},TL_{j},[s_{1},\cdots s_{k},\cdots s_{n}]\right\}$$

where $\left[s_{1},\cdots s_{k},\cdots s_{n}\right]$ represents the single-step fingerprints sequence of an atomic path. $TID_{j}$ represents the global long trajectory identifier. $T\theta_{j}$ represents the trajectory direction and $TL_{j}$ represents the step count of trajectory.

#### 3.3.3. Fingerprint Matching

**Figure 10:**
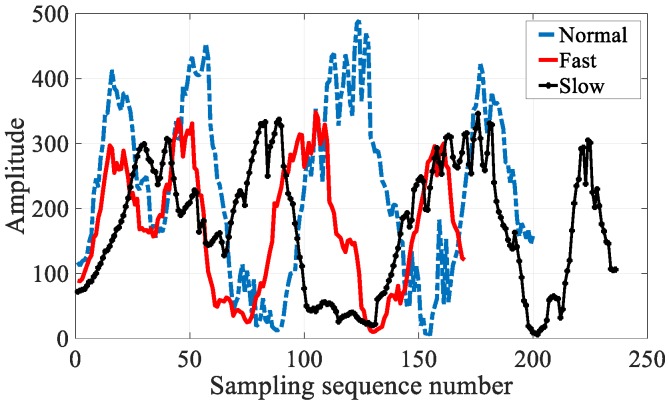
Different walking speed and sampling rate along a same path.

**Figure 11:**
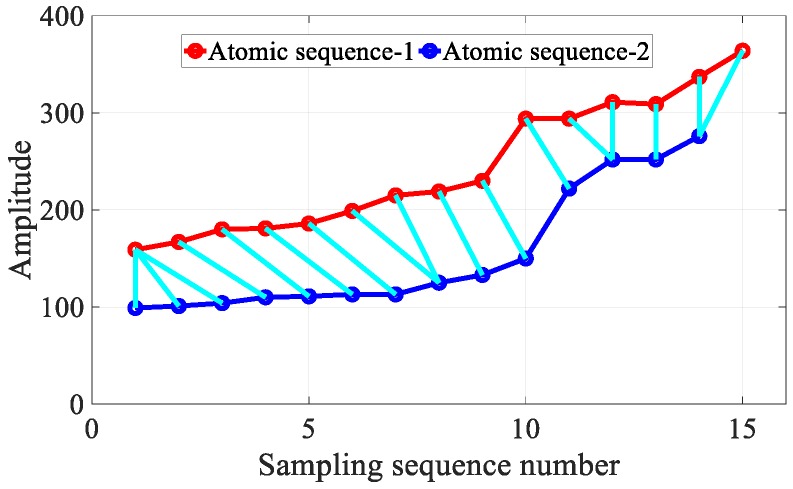
Atomic sequence matching using DTW.

As shown in Figure 10, different walking speeds result in the spatial sampling density variation issue. Fortunately, the DTW [39] algorithm from the automatic speech recognition field has the potential to align and measure the similarity between two time-series with different spatial sampling densities, owing to its robustness against series compression, stretching, and phase shifts, as shown in Figure 11. To eliminate the influence of different walking speeds and different sampling ratios, we leveraged the DTW algorithm to measure the similarity between two hybrid fingerprints sequences.

### 3.4. Single-Step Tracking Algorithm Based on Particle Filter Framework

The single-step tracking algorithm based on particle filter leverages a set of the particle to simulate all walking states of pedestrian, and the particle weight *w* represents the confidence of each walking state. A particle with higher weight means that it is closer to the actual position of pedestrian, and the weighted average of all particles’ positions is regarded as pedestrian’s position estimation.

Here, we describe a particle state as Equation (6):

(6) $$p_{t}^{i}=(x_{t}^{i},y_{t}^{i},\theta_{t}^{i},l_{t}^{i},w_{t}^{i})$$

where $p_{t}^{i}$ denotes the *i^th^* particle state at time *t*, $x_{t}^{i},y_{t}^{i}$ denote the position of the *i^th^* particle at time *t*, $w_{t}^{i}$ denotes the weight of the *i^th^* particle at time *t*, $\theta_{t}^{i}$ and $l_{t}^{i}$ represent the motion direction and distance of the *i^th^* particle at time *t*, respectively.

The particle filter framework contains four essential steps: (1) particle initialization. (2) Particle state prediction using the pedestrian motion model and floor plan constraints. Those particles moving into unreachable areas would be killed. (3) The weights of all particles are updated according to the DTW similarity between the latest observation fingerprints and trained fingerprints. (4) Particle resampling based on the weights of all particles. After initializing all particles, the particle filter iterates the step (2) to (4) to consistently track the target.

#### 3.4.1. Particle Initialization Based on Long Trajectory Matching

**Figure 12:**
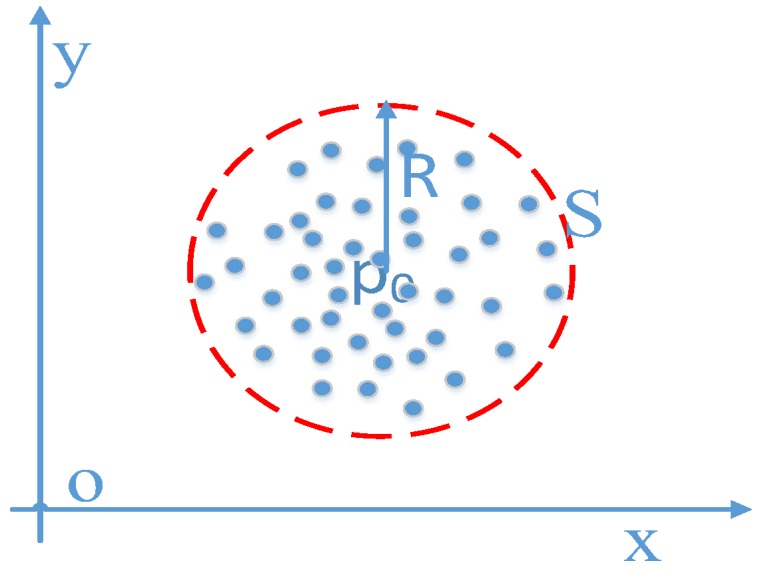
Particle initialization area based on long trajectory matching result.

As shown in Figure 12, the initial position of user *p*_0_ is determined by the result of LTM, and the particles are uniformly distributed in a circle *S* with center *p*_0_ and radius *R*. The weight of the particles is defined as $w_{0}^{t}=\frac{1}{N}\left(i=1,2,\ldots ,N\right)$, where *N* is the total number of particles.

#### 3.4.2. Motion Model

**Figure 13:**
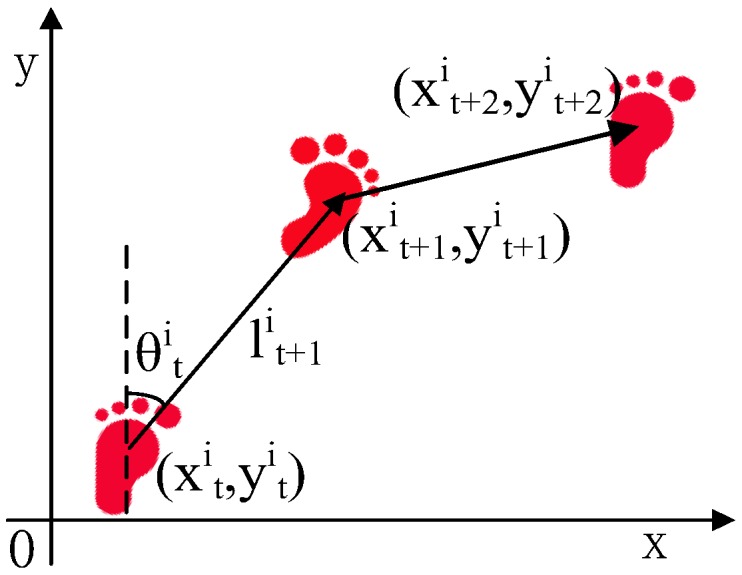
Motion model.

As shown in Equation (7) and Figure 13, we leveraged a pedometer to drive particle movement. Once the algorithm detects a step event, all particles move to new positions according to the heading estimation and step length of the latest step:

(7) $$\{\begin{array}{c} x_{\left(t+1\right)}^{i}=x_{t}^{i}+l_{\left(t+1\right)}^{i}\times \sin\theta_{\left(t+1\right)}^{i}\\ y_{\left(t+1\right)}^{i}=y_{t}^{i}+l_{\left(t+1\right)}^{i}\times \cos\theta_{\left(t+1\right)}^{i}\\ l_{\left(t+1\right)}^{i}=L_{t}^{}\times (1+G(0,\sigma_{l}^{2}))\\ \theta_{\left(t+1\right)}^{i}=\theta_{t}^{i}+\Delta \theta (1+G(0,\sigma_{\theta }^{2})) \end{array}$$

where Δ*θ* represents the heading change from the (*k*−1)*^th^* step to the *k^th^* step. Δ*θ* represents the heading obtained from the integral gyroscope. To enlarge the diversity of particles, we introduced Gaussian Noise *G*(0,$\sigma_{\theta }^{2}$) and *G*(0,$\sigma_{l}^{2}$) in the heading and step length estimation, respectively. Every person’s stride is different, and even the step length of the same person changes in different walking patterns. Inaccurate step length estimation leads to massive localization errors, and even to localization failure. Therefore, we employed a dynamic step length estimation to solve this challenge efficiently. We sorted the weight in descending order and chose the top 50% most weighted particles for dynamic step length estimation. *Lt* is the average step length of the former k-large particles weight at time *t*, which is defined as $L_{t}=\sum_{i=1}^{k}l_{t}^{i}g\omega_{t}^{i}/\sum_{i=1}^{k}\omega_{t}^{i}$. Compared with the step length estimation methods in [46] that are based on a turn detection algorithm that updates step length only when turning, our algorithm dynamically updates step length based on particle distribution in real time and is independent from the accuracy of the turning detection.

#### 3.4.3. Particle Constraints Based on the Floor Plan

**Figure 14:**
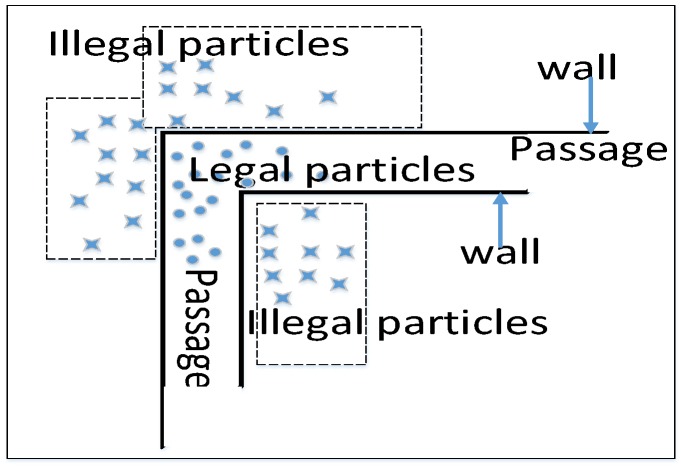
Diagram of particle constraints with the floor plan.

We constrained the particle motion range based on the floor plan that indicates the reachable areas (corridor, passage, hall) and the unreachable areas (walls, desks and other furniture). As shown in Figure 14, partial particles are across the wall. We called this kind of particles as irregular particles. We assumed that users would not go to the unreachable areas under normal circumstances. Therefore, all irregular particles’ weight will be set to 0 and we kill them.

#### 3.4.4. Weight Update Based on Single-Step Fingerprints

The weight of each particle is updated by the single-step fingerprints according to Equation (8):

(8) $$w_{t}^{i}=e^{-\frac{{\left(d_{m,t}^{i}\right)}^{2}}{2{\left(\sigma_{m,t}^{i}\right)}^{2}}}+e^{-\frac{{\left(d_{l,t}^{i}\right)}^{2}}{2{\left(\sigma_{l,t}^{i}\right)}^{2}}}$$

where $w_{t}^{i}$ represents the weight of the *i^th^* particle at time *t*. $d_{m,t}^{i}$ and $d_{l,t}^{i}$ represent *the DTW* distance of the magnetic field and light intensity between online and training single-step fingerprints, respectively. $\sigma_{m,t}^{i}$ and $\sigma_{l,t}^{i}$ represent the parameters that reflect the overall disturbance of the magnetic field and light intensity signals, respectively. After the weight of all particles was updated, we performed a normalization operation for all particles to maintain their sum equal to one before the resampling.

#### 3.4.5. Particle Resampling

Resampling aims to eliminate particles with a small weight that contribute little to estimating the location of a pedestrian, and concentrate on particles with a large weight that are much closer to the actual state. In this work, we generated new particles from old ones according to the particle state of the last round according to Equation (9). After a normalization operation, we sort all particles in the descending order by particles’ weights. A particle is cloned (i.e., resampled) according to which weight range the generated random number $y_{k}$ belonging to:

(9) $$\{\begin{array}{l} y^{k}=rand(0,1)\\ p^{k}=p^{i}\;\;\;\;\;if\;y^{k}\in [\sum_{j=1}^{i-1}\omega^{j},\sum_{j=1}^{i}\omega^{j}]\\ \omega^{k}=1/N\;\;\;\;\;i,k\in (1\cdots N) \end{array}$$

where *p^k^* denotes the *k^th^* particle state of current round. *p^i^* denotes the *i^th^* particle state of the last round. *y^k^* denotes the *k^th^* random number generated by a uniform distribution between 0 and 1. *ω^j^* is the weight of the *j^th^* particle of the last round. *N* is the total number of particles.

#### 3.4.6. Pedestrian Position Decision Strategy 

The distribution of particles reflects the likelihood of the pedestrian’s real position. The position *p* of pedestrian is estimated based on the weight *ω* of particles by Equation (10):

(10) $$p_{t}(x,y)=\{\begin{array}{cc} x_{t}^{i},y_{t}^{i} & \mathrm{particle}\;\mathrm{divergency},\;\mathrm{particle}\;\mathrm{with}\;\mathrm{maxinum}\;\mathrm{weight}\\ \sum_{i=0}^{k}\left(x_{t}^{i},y_{t}^{i}\right)\cdot w_{t}^{i}/\sum_{i=0}^{k}w_{t}^{i} & \mathrm{particle}\;\mathrm{converge} \end{array}.$$

If particles are diverging, we regard the position of the particle with maximum weight as the pedestrian position. We find pedestrian’s position quickly, but it is unstable. We perform a weighted average for the positions of all particles to achieve a more stable position when the particles are converged.

### 3.5. Long Trajectory Calibration Scheme Based on Undirected Weighted Graph Model

We collected users’ walking trajectories *T* = (*t_i_*, *i* = 1,2,L,*n*). Each walking trajectory $t_{i}$ is determined by two consecutive turns. Long trajectory calibration scheme utilized several single-step fingerprints to enhance the discernibility of hybrid fingerprints. In addition, the undirected weighted graph model was used to enhance matching accuracy and decrease the computational overhead of LTM. We designed several empirical criteria to identify which matching result is accurate. Lastly, we leveraged the accurate matching result to calibrate the heading and step length.

#### 3.5.1. Undirected Weighted Graph Model

**Figure 15:**
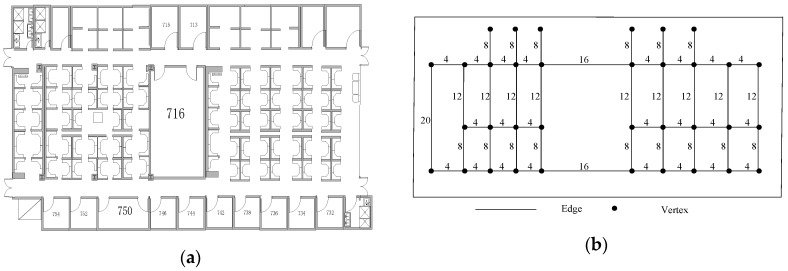
Undirected weighted graph model. (**a**) indoor floor-plan; (**b**) undirected weighted graph model.

As shown in Figure 15, the pathway information of the indoor floor-plan is modeled by the undirected trace-graph model. The vertexes represent the turning corners and the endings of pathways. The edges represent all pathways that users walk from one place to another. The edge is an atomic path including step number and Euclidean distance information between two vertexes. Here, the indoor floor-plan is denoted as *Graph*, which is described as:

(11) Graph={V,E}

where *V* is a coordinate set of all vertexes. If we use 2D coordinates to describe the indoor floor-plan, the set *V* is denoted as {(*x_i_*,*y_i_*), *i* =1,2,…,*N*}. Here, *N* represents the total number of vertexes. *E* represents a reachable pathways set of all vertexes, denoted as {(*w_p,q_*,*d_p,q_*), *p* = 1,2,…*N*, *q* = 1,2…,*N*}. Here *w_p,q_* denotes the number of steps between the *p^th^* and *q^th^* vertex. *d_p,q_* denotes the Euclidean distance between the *p^th^* and *q^th^* vertex.The undirected weighted graph model not only improves the efficiency of collection and reduces storage space of fingerprints, but also significantly reduces the computational overhead of LTM.

#### 3.5.2. Subsequence Matching

**Figure 16:**
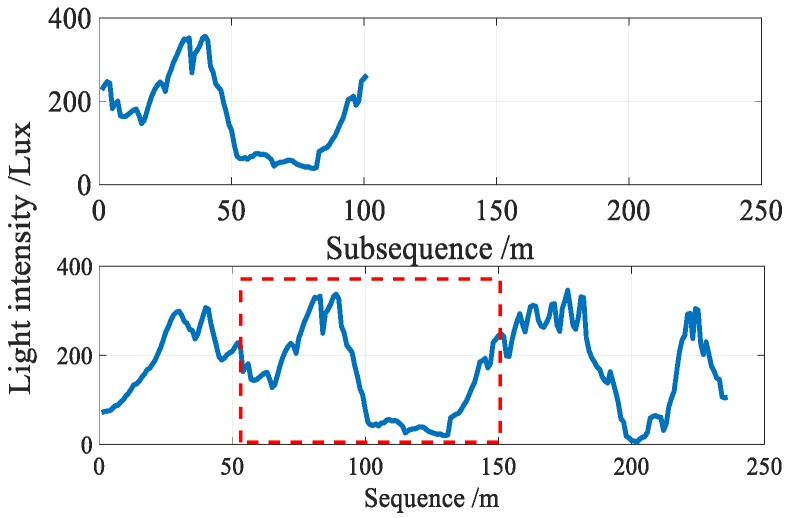
Long trajectory matching via Subsequence DTW.

In the process of real-time positioning, the user is usually in the middle of a trajectory and the input sequence is not complete. The online long trajectory sequence is usually much shorter than the training long trajectory sequences. We converted the DTW to subsequence DTW, which aligns and find the best matching long trajectory subsequence from all feasible training trajectories, as shown in Figure 16.

To reduce the computational overhead of LTM, we leveraged a sliding window mechanism to maintain an online sequence with fixed length. The sliding window matching mechanism triggers the matching operation immediately once the collected long trajectory fingerprints reach the predefined length, and then accumulates every step’s fingerprints data and performs the matching operation continuously.

#### 3.5.3. Long Trajectory Calibration Scheme

Algorithm 1 describes the procedure of the long trajectory calibration scheme, where *T* is the pre-trained trajectory set stored in a database, and t is the online trajectory. Once a perfect matching result is obtained, the single-step tracking result will be calibrated to an accurate location.

**Algorithm 1.** long trajectory calibration scheme1:**Input:** online trajectory t and training trajectory $T=\left\{t_{i},i=1,2,\cdots ,n\right\}$2:**Output:** user position $\left(x_{i},y_{i}\right)$3:Get the direction θ and length l of online trajectory t4:**If** the last step of t is turn **then**5:  Select similar trajectory according to θ and l6: **For** each training trajectory T **do**7:   Compute trajectory similarity degree $d_{i}$ between t and $t_{i}$ using DTW algorithm8:   Result validation9: **End for**10: **If** have perfect result **then**11:   Calibrate the result of single-step tracking algorithm12: **End if**13:
**End if**
14:**If** the last step of t is not turn, **then**15: Select similar trajectory according to θ16: **For** each training trajectory T **do**17: Compute trajectory similarity $d_{i}$ between t and $t_{i}$ using subsequence DTW algorithm18: Result validation19:
**End for**
20:**If** have perfect match result **then**21:   Calibrate the result of single-step tracking algorithm22:  **End if**23:
**End if**
24:
**Return**
$\left(x_{i},y_{i}\right)$


#### 3.5.4. Result Validation of Long Trajectory Matching

The DTW algorithm has the ability to align and find the most similar trajectory from all training trajectories. However, the most similar trajectory is not invariably a perfect trajectory. Therefore, we designed several empirical criteria to filter the DTW matching result and identify the correct matching result.

**Figure 17:**
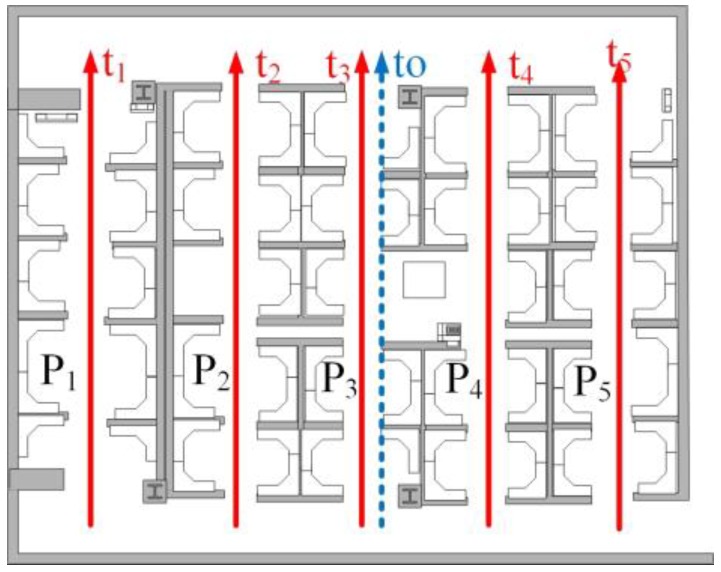
Spatial topology matching.

a) *DTW distance*. This quantity is calculated as the sum of the distances between the aligned samples normalized by the number of samples [8]. The normalized DTW distance between trajectory pairs of matched must be less than 5.
b) *Scale factor*. For each trajectory pair of matched $t_{a},t_{b}$ with $n_{a},n_{b}$ samples. The scale factor is sf=max(na,nb)min(na,nb), which represents how DTW stretches or compresses a trajectory to aligns and find the most similar trajectory. The scale factor must be less than 3. We assume that the speed of human walking differs little (commonly less than 3 times).
c) *Spatial topology*. The shape and space distance of matched trajectory in the PDR trace should also match. For each trajectory pair of matched *t_a_*,*t_b_*, we obtained two spatial coordinate vectors (*X_A_*,*Y_A_*) and (*X_B_*,*Y_B_*) $\left(X_{B},Y_{B}\right)$. After normalizing the coordinate of PDR trace pair to the origin of coordinates, the similarity of PDR trace is calculated by $\sum_{i=1}^{N}\sqrt{{\left(X_{Ai}^{}-X_{Bi}^{}\right)}^{2}+{\left(Y_{Ai}^{}-Y_{Bi}^{}\right)}^{2}}/N$. N denotes the number of the matched point pair after DTW stretching or compressing. The similarity of PDR trace must be less than an empirical threshold. As shown in Figure 17, five paths (*P*_1_,…,*P*_5_) correspond to five training trajectories (*t*_1_,…,*t*_5_) (in red). A pedestrian walks along *p*_3_, and obtains an online trajectory *t* (in blue). *d_i_* denotes the DTW distance between t and *t_i_*. The sensor noise may cause, *d*_1_<*d*_3_<min({*d*_2_,*d*_4_,*d*_5_}) thus mistaking *t*_1_ as the most similar trajectory. However, the space distance between *t*_1_ and *t*_0_ is much larger than that between *t*_3_ and *t*_0_. The spatial topology constraint can be used to filter out the incorrect mapping trajectory *t*_1_ and find correct trajectory *t*_3_ by the shape and space distance constraint of the PDR trace. Therefore, spatial topology filtering is an effective way to avoid mismatching.

#### 3.5.5. Adaptive Fusion of LTM Matching Results

We perform LTM algorithm for magnetic field fingerprints trajectory and light fingerprints trajectory, respectively. The confidence of LTM result is decided synthetically by the signal variance and the DTW distance. Therefore, the final pedestrian’s position estimation is estimated as follows:

(12) $$\left(x_{i},y_{i})=\frac{o_{l,i}}{o_{m,i}+o_{l,i}}\cdot (x_{m,i},y_{m,i})+\frac{o_{l,i}}{o_{m,i}+o_{l,i}}\cdot (x_{l,i},y_{l,i}\right)$$

(13) $$o_{l,i}=\frac{\sigma_{l,i}}{d_{l,i}},o_{m,i}=\frac{\sigma_{m,i}}{d_{m,i}}$$

where $\left(x_{m,i},y_{m,i}\right)$ and $\left(x_{l,i},y_{l,i}\right)$ denote the LTM result of magnetic field and light intensity, respectively. d represents the DTW distance in signal space for online and training trajectories. The larger distance indicates the lower similarity. σ denotes the variance of trajectory fingerprints. Larger variance indicates more signal features, and hence better matching results.

#### 3.5.6. Calibrating Heading and Step Length Based on the Undirected Weighted Graph Model

The subsequence DTW aligns and obtains the best-matched trajectory subsequence from the training trajectories. Once a perfect LTM result is obtained, the result of the single-step tracking algorithm is calibrated directly. In addition, the edge corresponding to the online trajectory is indexed from the UWGM. The Euclidean distance and direction of matched trajectory are then obtained according to the corresponding edge. The number of online steps from the last turn is counted by a pedometer. The length of online steps is calibrated by joining the step number of the online trajectory and the Euclidean distance of the matched trajectory. The pedestrian heading is also calibrated by the matched trajectory direction. Therefore, we obtained a more accurate pedestrian motion model by constantly updating both the step length and pedestrian heading. The long trajectory calibration scheme based on an undirected weighted graph model not only provides an initial position for the single-step tracking algorithm, but also improves the performance of the single-step tracking algorithm.

### 3.6. Other Considerations: Floor Identification

Identifying different floors in multistory buildings is an essential task for precise indoor localization, since a positioning system needs to load the floor plan and training model of the corresponding floor number before performing the single-step tracking algorithm and long trajectory calibration scheme. In this paper, we use a barometric pressure information-based floor identification algorithm [47] that identifies floors with more than 96.1% accuracy.

## 4. Experiments and Evaluation

To understand the effectiveness and limitations of LiMag, we implemented and evaluated LiMag in multiple complex indoor environments.

### 4.1. Experimental Setup

To fully evaluate the performance of LiMag, we conducted a full-fledged implementation based on a client-server architecture. The client side was an Android application that automatically collected sensors readings and displayed positioning results. The server hosted the database of hybrid fingerprints, collected the user uploaded fingerprints data and performed the computationally intensive positioning tasks. For each localization request, the server first loaded the floor plan and training data of the corresponding floor number, then found the best matching location of the phone, and returned the location to the client.

During the experiment, the data were collected by five participants (including three males and two females) within the height group of 158–193 cm using Android smartphones (one Huawei mate 9 with an 8 core 2.4 GHz processor and one Samsung S6 with a 4 Core 2.1 GHz and a 4 Core 1.5 GHz processor) in three typical scenarios (see Table 1). The smartphones were equipped with a 3-axis magnetic field sensor, light sensor, a 3-axis accelerometer, and a gyroscope. The smartphones periodically collected the data generated from the sensors mentioned above with a 20 Hz sampling rate.

To reduce the sampling cost and storage space, we propose a data collection method based on UWGM: the surveyor walked at a constant speed along these edges with the phone facing the ceiling to cover the entire area. The system automatically collected data captured by light and magnetometer built in smartphone during the walking. We calculated the location of the intermediate steps according to the step count and the overall distance of each edge. The location of each fingerprint was then interpolated from the locations of two consecutive steps. Finally, the hybrid fingerprint map was constructed by extrapolating the hybrid fingerprints on the survey path towards both sides until filling up the entire reachable area according to a floor plan that indicates the indoor layout, reachable areas and unreachable areas. We simply assumed pedestrian walking at 1 m/s. Eventually, we obtained hybrid fingerprint in every 0.05 m × 0.05 m square. Taking a 520 square meter office for example, the time to sample the hybrid fingerprint was less than 15 min for one person. The total walking distance was 280 m (448 steps). The size of the hybrid fingerprint was about 1 M.

### 4.2. Localization Accuracy in Typical Scenarios

**Figure 18:**
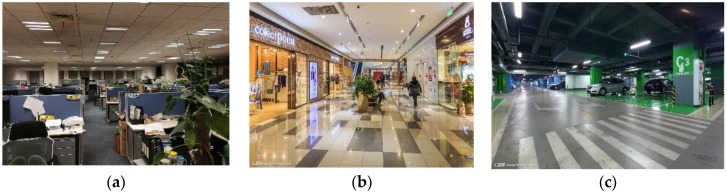
Typical indoor scenarios. (**a**) Office; (**b**) Shopping mall; (**c**) Parking lot.

**Figure 19:**
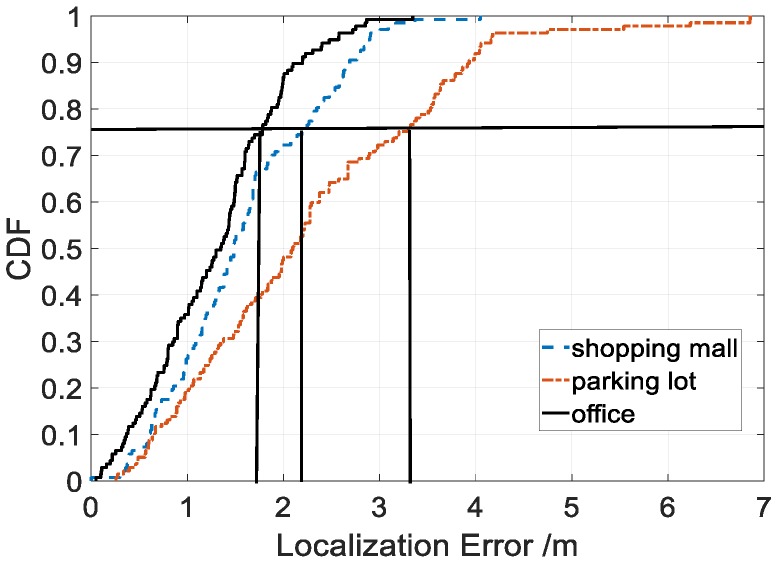
Localization accuracy in typical scenarios.

To verify the performance and practicality of the proposed method, we conducted extensive experiments in three typical scenarios: offices, shopping malls, and underground parking lots, see Figure 18. We walked randomly in each scenario and collected hybrid fingerprints of light intensity and magnetic field during walks. We then ran LiMag to estimate user positions and calculate the localization errors. As shown in Figure 19, the cumulative distribution function (CDF) of localization errors demonstrated that our proposed algorithm achieved 75th percentile localization accuracy of 1.8 m, 2.2 m and 3.3 m in offices, shopping malls and parking lots, respectively. Table 2 shows that the average localization error using hybrid fingerprints was 1.29 m, 1.58 m and 2.26 m in the offices, shopping malls and parking lots, respectively. Compared with offices and shopping malls, the standard deviation of the localization error in parking lots was larger. Among the three typical scenarios, the localization accuracy of the parking lots was relatively poor, since non-evident optical features were caused by the sparsely-deployed fluorescent lamps and unstable magnetic field distribution caused by movement of the vehicles.

### 4.3. Localization Accuracy in Sunlight Interference Scenario

**Figure 20:**
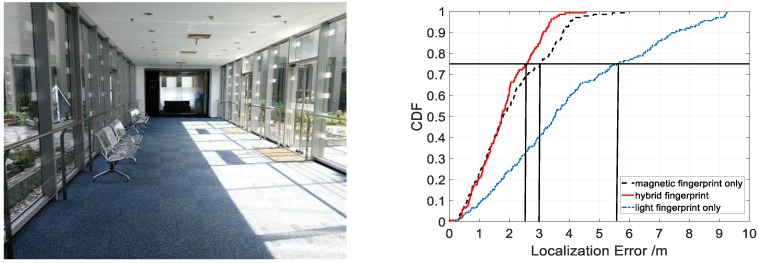
Localization accuracy of LiMag using different fingerprint types in sunlight interference areas.

To validate the anti-interference ability of LiMag, we conducted experiments in buildings with large windows and skylights. The localization performance in CDF form from our proposed algorithm using different fingerprint types is shown in Figure 20. The results demonstrated that our proposed algorithm achieved 75th percentile localization accuracy of 2.5 m, 3 m and 5.6 m using hybrid, magnetic field and light intensity fingerprint in an environment characterized by strong sunlight, respectively. The hybrid fingerprints of light and magnetism achieved 3 m localization accuracy with about 85% confidence, while the light fingerprints only achieved 3 m localization error only with 40% confidence. Table 3 shows that the average localization error was 1.83 m, 3.95 m and 2.05 m in an environment characterized by strong sunlight using hybrid fingerprints, light fingerprints only and magnetic fingerprints only, respectively. Compared with the magnetic fingerprints only and hybrid fingerprints, the standard deviation of the localization error using light fingerprints was larger. As shown in Figure 20 and Table 3, the positioning performance of light fingerprints only decreased dramatically when the user entered the sunlight interference area because the sunlight disturbs the light sensor readings severely in sunlight interference area. The result indicated that the hybrid fingerprints improved the localization accuracy evidently in an environment characterized by strong sunlight because the magnetic signal is not affected by the sunlight. We fully utilized the advantages of the magnetic field and light intensity to provide a more accurate location service.

### 4.4. Localization Accuracy in Open-Plan Areas

**Figure 21:**
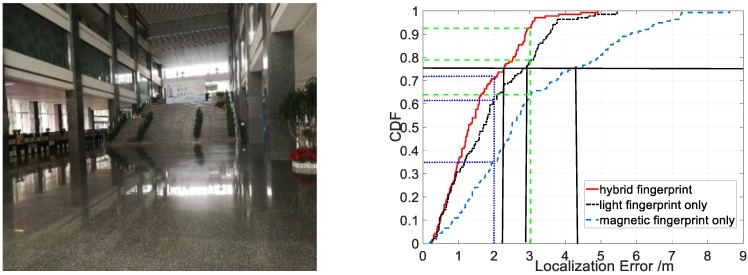
Localization accuracy of LiMag using different types of fingerprints in open-plan area.

To evaluate the localization accuracy of the open-plan area, we conducted experiments in broad and open plan environments. Figure 21 exhibits the localization error in CDF form from our proposed algorithm using light fingerprint, magnetic fingerprint, hybrid fingerprints of light and magnetic. The results demonstrated that our proposed algorithm achieved 75th percentile localization accuracy of 2.3 m 4.4 m and 2.9 m using hybrid, magnetic and light fingerprint in the open-plan area, respectively. The hybrid fingerprints of light and magnetism achieved 2 m localization error with about 72% confidence, while the magnetic fingerprints only achieved 2 m localization error only with 35% confidence. The hybrid fingerprints of light and magnetism achieved 3 m localization error with about 92% confidence, while the magnetic fingerprints only achieved 3 m localization error only with 64% confidence. Table 4 shows that the average localization error was 1.55 m, 1.88 m and 2.99 m in the open-plan area using hybrid fingerprints, the light fingerprints only and magnetic fingerprints only, respectively. Compared with the light fingerprints only and magnetic fingerprints only, the standard deviation of the localization error using and hybrid fingerprints of light magnetism was also substantially smaller. In other words, the feeble magnetic signal distortion in broad and open plan environments resulted in that localization accuracy solely based on magnetic fingerprints was inaccurate. The visible light improved the localization accuracy evidently in the open-plan area.

### 4.5. Localization Accuracy vs. Trajectory Length

**Figure 22:**
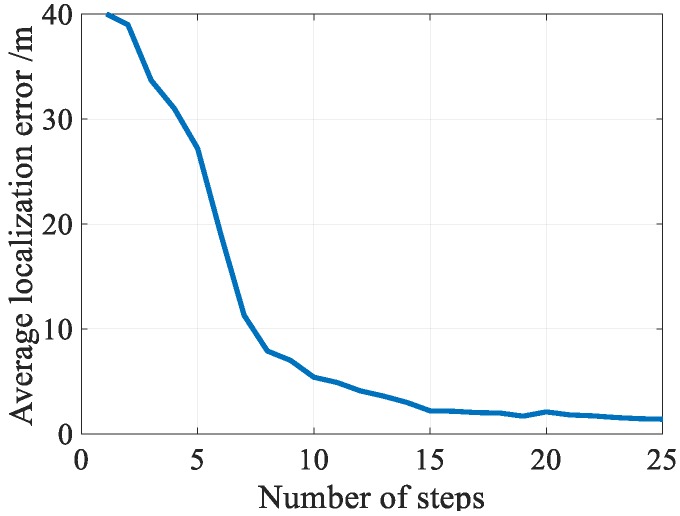
Matching accuracy vs. trajectory length.

Location differentiation ability of trajectory fingerprints depends on the length of trajectory fingerprints. To evaluate how the length of trajectory affects the localization performance of the long trajectory calibration scheme, and find the best trajectory length, we conducted extensive experiments on long trajectory calibration scheme with various length of trajectory. Figure 22 shows the matching errors corresponding to different lengths of trajectory. From the figure, we saw that the matching errors decreased with an increase in trajectory length. However, increasing the trajectory length will greatly increase the computational complexity and reduce the number of trigger calibrations. To balance the contradiction between matching error and matching number, the trajectory length was set to 15 steps in this paper.

## 5. Conclusions

In this work, we have presented an infrastructure-free indoor positioning system based on smartphone user using ubiquitous magnetic field and arbitrary commercial off-the-shelf (COTS) lamps that already exist in today’s buildings. LiMag constructed an HFM using magnetic field and light intensity information, and achieved real-time positioning and tracking based on HFM and particle filter. To accelerate the particle convergence and eliminate the accumulative error of our single-step tracking algorithm based on a particle filter framework, a long trajectory calibration scheme based on UWGM was designed. In addition to typical indoor scenarios, including offices, shopping malls and parking lots, we also conducted experiments in challenging scenarios including large open-plan areas and environments characterized by strong sunlight. The results demonstrated that LiMag provides location-based service with high accuracy, infrastructure-free, practically, as well as satisfactory robustness inside complex indoor environments.

## Figures and Tables

**Table 1:** Three typical scenario description.

		Office	Parking	Shopping Mall
Lamp	Deployment	Regular	Irregular	Irregular
	Type	CFL,LFL	ILB	CFL,LFL,LED
	Number	362	120	660
	Interval	0.3–2 m	4–8 m	1–3 m
Building	Area	520 m^2^	640 m^2^	1800 m^2^
	Type	Concrete	Concrete	Concrete
	Sunlight	Lots of windows	No windows	Lots of windows and skylights
	Electronic instrument	Lots of computers	No	Little computers
	Iron shelf	Little shelves	No	Lots of shelves

**Table 2:** The localization error in typical scenarios using hybrid fingerprints.

Fingerprint Type	Mean Localization Error	Standard Deviation of Localization Error
Office	1.29 m	0.69 m
Shopping mall	1.58 m	0.79 m
Parking lot	2.26 m	1.32 m

**Table 3:** The localization error in sunlight interference scenario using different fingerprints.

Fingerprint Type	Mean Localization Error	Standard Deviation of Localization Error
Light fingerprints	3.95 m	2.39 m
Magnetic field fingerprints	2.05 m	1.22 m
Hybrid fingerprints	1.83 m	0.93 m

**Table 4:** The localization error in the open-plan area using different fingerprints.

Fingerprint Type	Mean Localization Error	Standard Deviation of Localization Error
Light fingerprints	1.88 m	1.19 m
Magnetic field fingerprints	2.99 m	1.88 m
Hybrid fingerprints	1.55 m	0.94 m
